# Thumb reconstruction after radical tumor resection using free osteocutaneous lateral arm flap with secondary humerus fracture—a case report

**DOI:** 10.1007/s00402-022-04623-x

**Published:** 2022-09-23

**Authors:** Judy Martin, Feras Taqatqeh, Adrian Dragu, Dmitry Notov, Hagen Fritzsche, Oana Grigorescu, Olimpiu Bota

**Affiliations:** Faculty of Medicine Carl Gustav Carus, University Center for Orthopedics, Trauma and Plastic Surgery, TU Dresden, Fetscherstraße 74, 01307 Dresden, Germany

**Keywords:** Thumb reconstruction, Lateral arm flap, Free osteocutaneous flap, Tumor, Squamous cell carcinoma

## Abstract

**Introduction:**

Malignant diseases with infiltration of bony structures in the area of the phalanges or metacarpals require either amputation or complex reconstruction. The decision for reconstruction means to restore length, mobility, sensibility, stability as well as aesthetics.

**Methods:**

We present a case of complex first ray reconstruction of the left hand using a free osteocutaneous lateral arm flap from the ipsilateral side. The reconstruction was performed after radical resection of an exulcerated squamous cell carcinoma, including the first metacarpal bone, trapezium, partial trapezoid and distal scaphoid as well as partial resection of the extensor pollicis longus, extensor pollicis brevis, abductor pollicis longus and flexor carpi radialis tendons. The osteosynthetic restoration was achieved distally by a double wire cerclage and a proximally by temporary K-wire suspension. Moreover, to reconstruct the extensor pollicis longus tendon the ipsilateral palmaris longus tendon was harvested and used. Postoperatively, a secondary humerus fracture occurred, which was initially attended by plate osteosynthesis. The fracture showed delayed healing, which was treated by re-plating and autologous cancellous bone.

**Results:**

12 months postoperatively, the patient showed an excellent outcome with length preservation and good range of motion, sensibility, stability and aesthetic of the thumb. Furthermore, the quarterly tumor aftercare showed no evidence of recurrence.

**Conclusion:**

This case report showed that the free osteocutaneous lateral arm flap is a reliable solution for the reconstruction of the first ray with a great functional and aesthetic outcome. To prevent a secondary humerus fracture, a preventive plate osteosynthesis simultaneous with the osteocutaneous flap elevation should be considered.

**Supplementary Information:**

The online version contains supplementary material available at 10.1007/s00402-022-04623-x.

## Introduction

The squamous cell carcinoma (SCC) of the skin arises from degenerated epidermal keratinocytes. Currently, no effective standardized drug therapy is available, so the highest healing rate is achieved with a surgical R0 resection leaving extensive defects [1]. Combined carpal bone and soft tissue defects require a complex microvascular reconstruction with appropriate expertise to maintain the organ. The free lateral arm flap (LAF) was first described by Song et al. in 1982 and can be elevated as cutaneous, fasciocutaneous or osteocutaneous either pedicled or free flap. The vascular supply is ensured by the posterior radial collateral artery (PRCA) via lateral intermuscular septum. Additionally, the bone receives its supply from 1 to 2 branches of the PRCA which often appear 3–6 cm proximal to the lateral epicondyle [2].

### Case report

**Fig. 1:**
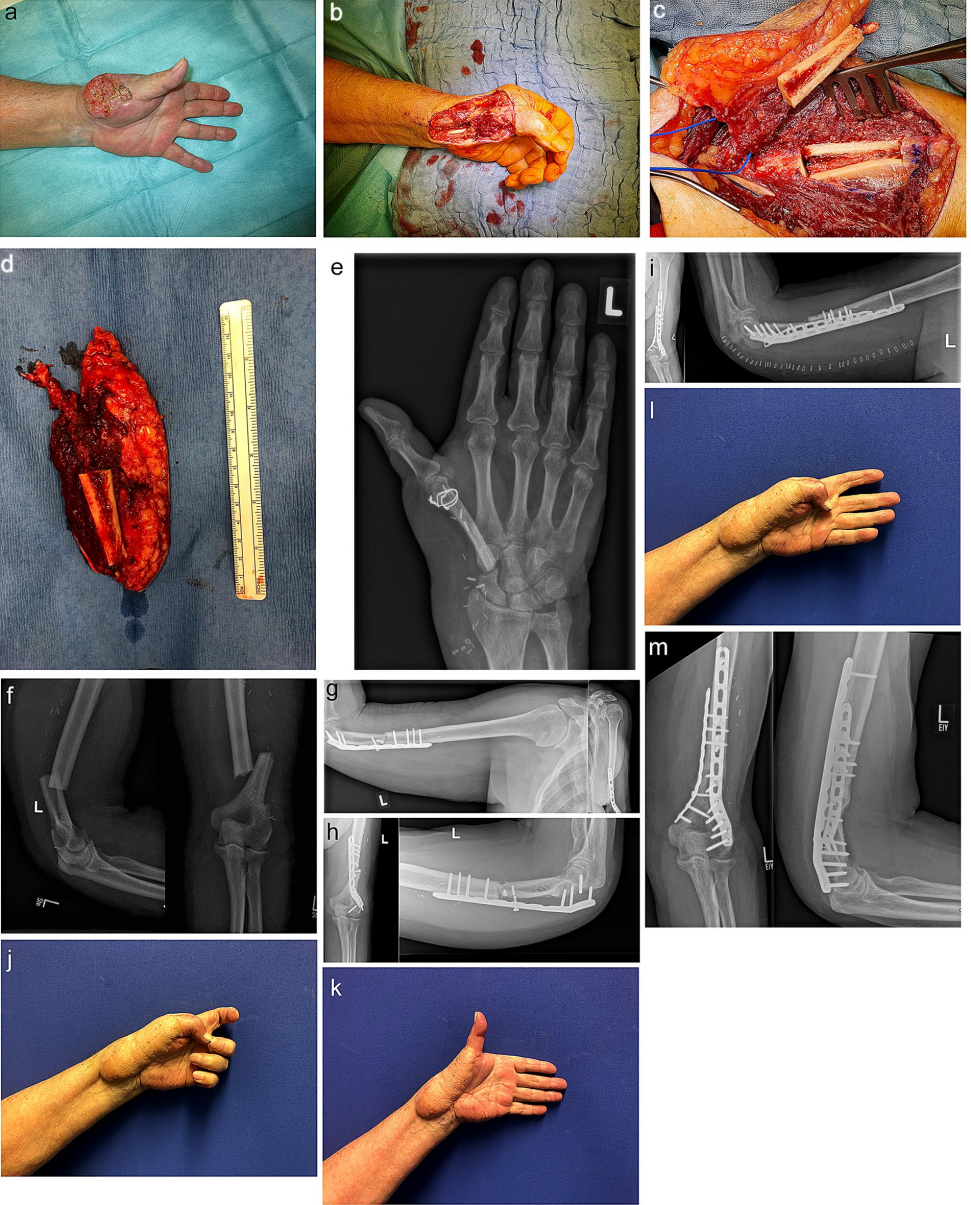
**a** Before radical resection. **b** After radical resection. **c** Free osteocutaneous LAF before elevation. **d** Free osteocutaneous LAF after elevation. **e** X-ray 12 months after initial operation. **f** Humerus fracture. **g** Humerus fracture after plate osteosynthesis. **h** Plate and screw dislocation after initial osteosynthesis. **i** X-Ray after cancellous bone transplantation. **j**–**l** Final result achieved 12 months after reconstruction. **m** X-Ray 7 months after resection of the pseudarthrosis and spongioplasty

A 72-year-old man presented with an exulcerated SCC of the left thumb with infiltration of soft tissues, shaft and base of first metacarpal bone, trapezium, trapezoid and distal scaphoid (Fig. 1a). After a detailed clinical examination, preoperative staging diagnostics, which included surgical sampling and histological examination, X-rays and MRI of the left hand, sonography of the axillary lymph nodes and tumor board review were performed. The followed radical en bloc resection encompassed the shaft and base of the first metacarpal bone, trapezium, the radial half of trapezoid and distal scaphoid as well as extensor pollicis longus (EPL), extensor pollicis brevis, abductor pollicis longus and flexor carpi radialis tendons and a thenar muscle cuff. The histopathological result showed a R-1 situation at the palmar cutaneous and subcutaneous edge of the wound. After further resection and histological confirmation of an R-0 situation, the reconstruction was performed (Fig. 1b).

First, a high arm torniquet with 280 mmHg was applied to the ipsilateral arm. Flap preparation proceeded laterally, with identification of the intermuscular septum. A muscle cuff with a 5 mm triceps tendon strip were simultaneously elevated. Then the dissection continued medially, with isolation and protection of the radial nerve. The septum was isolated together with a vascularised fragment of the humerus bone 3 cm above the lateral epicondyle, 5 cm long and 1.0 × 1.5 cm wide (Fig. 1c, d). The medial collateral artery was prepared distally over 2 cm and elevated together with the posterior radial collateral artery and the accompanying veins as a T-piece for a flow-through flap.

The flap was brought to the hand defect. First, the first metacarpal was restored using the humeral bone fragment. Distally, the remaining metacarpal head was united to the humeral bone fragment with two perpendicular wire cerclages (Fig. 1e). Proximally, the carpometacarpal joint was reconstructed by suspending the bone fragment to the second and third metacarpal with a 1,2 mm k-wire and by filling the trapezium compartment with triceps muscle as an interpositional arthroplasty. The elevated triceps tendon was not sufficient for an EPL reconstruction, hence the reconstruction of the EPL tendon was carried out by harvesting the palmaris longus tendon.

As recipient vessels, the radial artery and two accompanying veins as well as the cephalic vein were prepared under the microscope. The radial artery was severed and the proximal end was anastomosed with the posterior radial collateral artery, while the distal end was anastomosed with the medial collateral artery as a flow-through flap. The two accompanying veins and the cephalic vein were connected using vein couplers. The soft tissue defect could be closed without tension, with the proximal part covering the vessels anastomoses.

An intensive postoperative flap monitoring regime was initiated, with simultaneous lymphatic drainage of the hand. After 7 days, a forearm thumb cast was installed. Five weeks postoperatively, the k-wire was removed, as consolidation was seen on the X-Ray and full mobilization of the thumb with physiotherapy assistance was initiated.

On the fifth postoperative day, a secondary humerus fracture occurred in the context of patient mobilization, which was initially treated by dorsal plate osteosynthesis. Five months later, a delayed union of the bone with dislocation of the plate and screws was noted. A revisional operation stabilized the humerus with two locking compression plates and autologous cancellous bone (Fig. 1f–i). No neurological deficits of the radial or ulnar nerve were noted during the treatment.

The final examination 12 months postoperatively showed an excellent range of motion with five points on the Kapandji opposition score (Fig. 1d) (Online resource 1), a flexion and extension in the IP joint of (86°/0°/20°) and in the MCP joint of (40°/0°/12°), an abduction in the hand plane of 70° and orthogonal to the hand plane of 60° (Fig. 1j–l) (Online resource 2). A satisfactory outcome was achieved, including length preservation, good sensibility (two-point-discrimination at the tip of the thumb of 5 mm), stability and aesthetic of the thumb. The quarterly tumor aftercare including ultrasound of the hand and regional lymph nodes showed no evidence of recurrence. Currently, 7 months after revision of the humeral fracture, a good bony consolidation and a ROM of (0/0/150) were achieved (Fig. 1m). The scar on the upper arm was completely asymptomatic and inconspicuous. 

### Discussion

Approximately, 70 percent of soft tissue tumors occurring on the hand are SCC, which have the tendency to infiltrate subcutaneous tissues, vessels, nerve sheaths, tendons, cartilage, and bone [3]. In the case of periosteal infiltration, a tumor size greater than 20 mm or the presence of satellite lesions, an amputation is commonly indicated, which results in corresponding functional and aesthetic restrictions [4].

In our case, we present the reconstruction of a complex bony, tendon and soft tissue defect of the first ray after radical tumor resection. The indication to preserve the thumb was established firstly on the importance of the thumb function, secondly on the presence of intact palmar soft tissues, especially of the sensitive fingertip and thirdly on the presence of the metacarpophalangeal joint. Therefore, we planned a complex reconstruction using a free composite osteocutaneous LAF to salvage the left thumb. The composite osteocutaneous LAF is of particular importance due to the ability to perform a simultaneous complex osseous and cutaneous defect reconstruction by harvesting a vascularised fragment of the humerus bone. It is indicated for reconstructions of the hand, thumb and lower extremity with combined bone and soft tissue defects [2]

In addition to individual factors, Kremer et al. indicate osteocutaneous fibula grafts for defects above 8 cm and osteomyocutaneous flaps from the subscapular system for defects between 4 and 8 cm as first choice [5]. Accordingly, the free osteocutaneous LAF is only suitable for smaller defects. We used a free LAF with a bone fragment measuring 5 cm in length for thumb reconstruction showing marvelous results. Furthermore, Arnež et al. [6] state the possible use of a 10 cm bony fragment when elevating the LAF.

Haas et al. [7] mention two cases in which a reconstruction using an osteocutaneous LAF was carried out after traumatic thumb injuries. In one of the cases, the IP joint was uninjured and the EPL tendon was reconstructed using an interposing triceps tendon. Generally, extensor indices transfer or a free tendon graft using the palmaris longus (PT) tendon are common options for reconstructing the EPL tendon. In our case, the defect was too large for the triceps tendon to be used. Therefore, the ipsilateral palmaris longus tendon was harvested, in order to avoid further damage in the area by harvesting the extensor indices proprius (EIP) tendon. The downside of PT transfer is a higher risk of infection or adhesions. In our case, the strong Pulvertaft tendon suture allowed an early mobilization with physiotherapy, avoiding contractures, while the well vascularized free flap protected the PT tendon against infection due to the contaminated wound. As opposed to the EIP transfer, the uninjured motor units are preserved and retraining is not necessary [8]. We found that reconstructing the EPL tendon, the thumb extension as well as the abduction would be restored, so did not reconstruct the remaining APL and EPB tendons [9]

The most common surgical treatment of the carpometacarpal joint of the thumb implies trapezium resection. Usually, proximalization of the first metacarpal with impingement on the scaphoid is prevented by the intermetacarpal ligament, which in this case was resected with the first metacarpal base. We suspended the bone fragment to the remaining metacarpal bases and interposed the triceps muscle, hence providing an original suspension arthroplasty. The new metacarpal bone was retained in place, while the triceps muscle could fibrose.

Arnež et al. [6] reported a serie of three cases of extended osteocutaneous sensory free LAF for posttraumatic thumb reconstruction, with satisfactory results. To prevent secondary humerus fractures Arnež et al. [6] defined the maximum diameter of the extracted humerus fragment with 1.0 × 1.5 cm. Windhofer et al. [10] reported four cases of forearm reconstruction using a free LAF without complications. For prevention of fractures Windhofer et al. supplied all patients with a locking compression condyle plate simultaneously [10]. In our case a secondary humerus fracture occurred at the level of the proximal osteotomy, without an adequate trauma. As the size of the harvested bone was within the recommended dimensions, a preventive plate osteosynthesis simultaneous with the osteocutaneous flap elevation should be considered. We attribute the delayed union to the transverse cortical fracture in an elderly patient with reduced bone contact after reduction of the humeral diameter. The revisional surgery supplied more stability through the double plate osteosynthesis as well as osteogenic cancellous bone and therefore ensured the bone healing.

One limitation of the reported technique would be the donor site morbidity, consequent to any autologous reconstruction. A strict benefit and cost analysis is essential. Furthermore, optimized early diagnosis could minimize the extent of resection and new approaches in tissue engineering could preclude the need for autologous tissue harvesting and therefore reduce the donor site morbidity in the future.

In conclusion, our case reveals that a free osteocutaneous LAF for thumb reconstruction can be a useful tool for reconstructing bone and soft tissue while preserving length with an extraordinary mobility, sensibility, stability as well as aesthetic of the thumb.

## Supplementary Information

Below is the link to the electronic supplementary material.

**Online resource 1: **Range of motion 12 months postoperatively with 5 points on the Kapandji opposition score (MP4 4260 KB)

**Online resource 2: **Range of motion 12 months postoperatively (MP4 2985 KB)

## References

[1] Brancaccio G, Briatico G, Pellegrini C (2021). Risk factors and diagnosis of advanced cutaneous squamous cell carcinoma. Dermatol Pract Concept.

[2] Kokkalis ZT, Papanikos E, Mazis GA (2019). Lateral arm flap: indications and techniques. Eur J Orthop Surg Traumatol.

[3] Wollina U, Tempel S, Albert W (2019). Advanced ulcerated squamous cell carcinoma of the hand with locoregional axillary lymph node metastasis—case report and literature review. Open Access Maced J Med Sci.

[4] Tripoli M, Cordova A, Moschella F (2017). Characteristics, management techniques, and outcomes of the most common soft-tissue hand tumors: a literature review and our experience. Ann Plast Surg.

[5] Kremer T, Bickert B, Germann G (2006). Outcome assessment after reconstruction of complex defects of the forearm and hand with osteocutaneous free flaps. Plast Reconstr Surg.

[6] Arnež ZM, Kersnič M, Smith RW (1991). Free lateral arm osteocutaneous neurosensory flap for thumb reconstruction. Journal of Hand Surgery.

[7] Haas F, Rappl T, Koch H (2003). Free osteocutaneous lateral arm flap: anatomy and clinical applications. Microsurgery.

[8] Meiwandi A, Kaptanis S, Papadakis M (2020). Extensor indicis transfer versus palmaris longus transplantation in reconstruction of extensor pollicis longus tendon: a protocol for a systematic review. Syst Rev.

[9] Colantoni Woodside J, Bindra RR (2015). Rerouting extensor pollicis longus tendon transfer. J Hand Surg Am.

[10] Windhofer C, Michlits W, Karlbauer A (2011). Treatment of segmental bone and soft-tissue defects of the forearm with the free osteocutaneous lateral arm flap. J Trauma.

